# Supplementary material for: Diagnosis of Ion-Exchange Resin Depositions in Paraffin Sections Using Corrective Light and Electron Microscopy-NanoSuit Method
Source: Diagnostics (Basel). 2021 Jun 30;11(7):1193. doi: 10.3390/diagnostics11071193 (PMC8304092; doi:10.3390/diagnostics11071193)
Supplement: Supplementary file 1 [file diagnostics-11-01193-s001.zip › diagnostics-1267814-supplementary.pdf]

Supplemental Material

Supplementary Table S1. List of weight percentage (wt%) of elements detected by SEM-EDS analysis using the NanoSuit-CLEM method.

		C	O	S	K	P	La	N
Patient 1	deposition	39.1	41.48	17.16	1.9	0.35	0	0
	background	46.88	47.37	1.13	1.14	1.52	0.06	1.9
Patient 2	deposition	52.76	25.04	21.69	0.18	0.34	0	0
	background	59.94	29.52	1.26	0.94	0.66	0	7.68
Patient 3	deposition	53.56	31.63	14.01	0.25	0.41	0.13	0
	background	52.91	36.09	0.81	0.5	0.94	0	8.75
Patient 4	deposition	51.47	27.75	19.87	0.27	0.44	0	0.2
	background	46.24	44.04	0.89	0.79	0.85	0.38	6.81
Patient 5	deposition	26.16	32.21	0.39	0.32	8.26	32.65	0
	background	54.59	32.12	0.74	0.21	1.48	0.23	10.73
Patient 6	deposition	53.71	22.56	21.41	0.8	0.66	0	0.86
	background	51.75	36.03	0.42	0.98	1.35	0	9.48
Pateint 7	deposition	49.66	25.65	21.61	0.3	0.44	0.09	2.24
	background	55.58	35.59	1.13	1.54	3.59	0	2.56
Patient 8	deposition	39.25	35.12	24.22	0.55	0.8	0.05	0
	background	44.7	48.4	0.26	1.49	1.42	0.39	3.29
Patient 9	deposition	47.61	36.37	15.43	0.25	0.34	0	0
	background	48.87	41.22	0.43	0.56	0.47	0	8.46

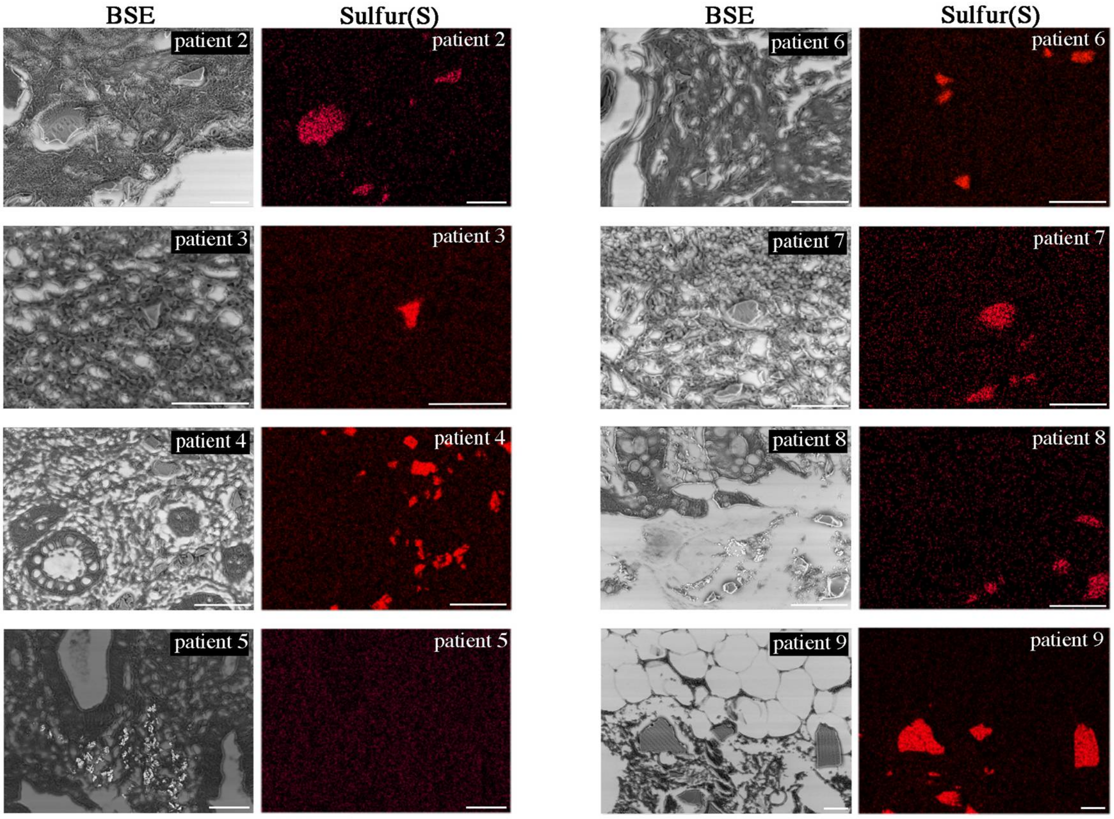

Supplementary Figure S1. The results detecting sulfur deposition in the gastrointestinal mucosa by SEM-EDS analysis using the NanoSuit-CLEM method (cases 2–9). Images in the left column are back-scattered electron (BSE) SEM images and images in the right column are EDS maps of sulfur. Red color represents sulfur deposition. White bar, 50 μm.

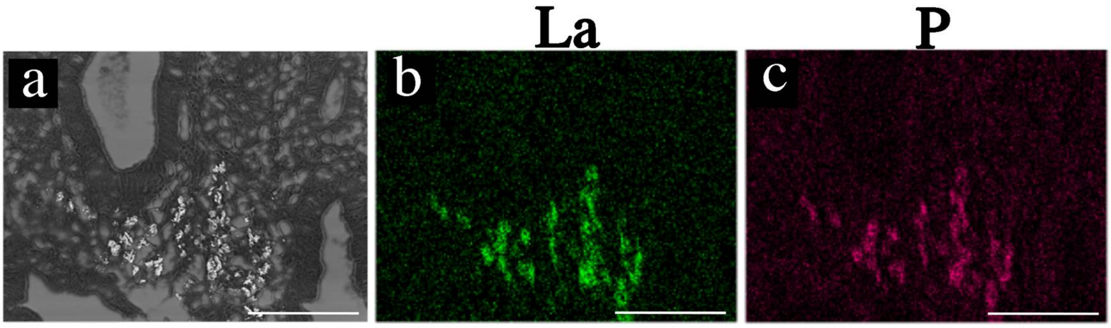

Supplementary Figure S2. The results detecting lanthanum and phosphorus deposition in the gastric mucosa by SEM-EDS analysis using the NanoSuit-CLEM method (case 5). Images in the left column are backscattered SEM images showing a bright area in the mucosa. The middle- and right-column images are elemental mapping images using SEM-EDS analysis showing the deposition of lanthanum (La) and phosphorus (P), respectively. White bars represent 100 μm.

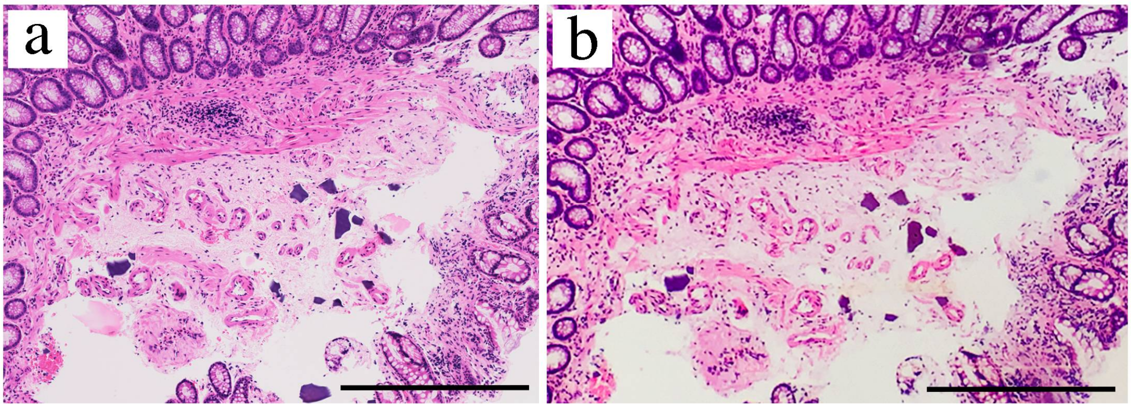

Supplementary Figure S3. Comparison of H&E staining between pre- and post-EDS analysis. H&E staining of (**a**) pre- and (**b**) post-EDS analysis. Bars represent 50 μm

Supplementary Data 1. Comparison with weight percentage (wt%) of carbon and phosphorous detected between Sevelamer and Cholestyramine.

Sevelamer				Cholestyramine		
C(weight %)	P(weight %)	P/C		C(weight %)	P(weight %)	P/C
82.19	17.81	0.21669303		93.13	6.87	0.07376785
81.8	18.2	0.22249389		91.22	8.78	0.09625082
74.83	25.17	0.33636242		88.52	11.48	0.12968821
89.27	10.78	0.12075725		89.29	10.71	0.11994624
81.1	18.9	0.23304562		92.16	7.84	0.08506944
86.74	13.26	0.15287065		96.52	3.48	0.0360547
85.13	14.83	0.17420416		93.34	6.66	0.07135205
82.49	17.51	0.21226815		92.79	7.21	0.07770234
83.77	16.23	0.19374478		88.16	11.84	0.13430127
81.96	18.04	0.22010737		95.41	4.59	0.04810816
81.49	18.51	0.22714443		92.4	5.8	0.06277056
84.84	15.16	0.1786893		96.96	3.04	0.03135314
86.76	13.24	0.15260489		96.39	3.61	0.03745202
77.96	22.04	0.28270908		96.67	3.33	0.03444709
84.18	15.82	0.18793062		96.56	3.44	0.03562552
84.8	15.2	0.17924528		92.42	7.58	0.08201688
84.15	15.85	0.18835413		92	8	0.08695652
82.79	17.21	0.20787535		91.22	8.78	0.09625082
80.59	19.41	0.24084874		96.14	3.86	0.04014978
83.34	16.66	0.19990401		92.43	7.57	0.08189982
